# Gastric Outlet Obstruction Caused by a Foreign Body Ingestion

**DOI:** 10.1097/PG9.0000000000000249

**Published:** 2022-12-02

**Authors:** Submitted by:, Lea Oliveros, Michelle Rosario, Alexander Wilsey, Sara Karjoo, Michael Wilsey

**Affiliations:** Gastroenterology and Hepatology Fellowship, Penn State Health Milton S. Hershey Medical Center, Hershey, Pennsylvania, Department of Pediatric Gastroenterology, Johns Hopkins All Children’s Hospital, Saint Petersburg, Florida, Johns Hopkins All Children’s Hospital, Saint Petersburg, Florida

**FIGURE 1.**
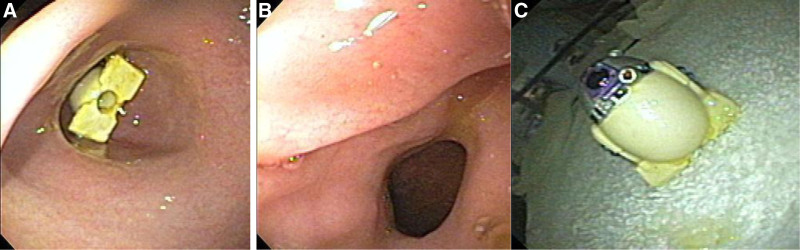
A) R2-D2 children’s toy lodged in the pylorus. B) Pylorus after removal of the toy. C) Angry Birds R2-D2 Egg after extraction from the patient’s gastrointestinal tract.

After swallowing his plastic R2-D2 Angry Birds Egg (4.3 × 3.2 × 2.1 cm), a 4-year-old male had been asymptomatic overnight, but the following morning he developed intractable, nonbloody, nonbilious emesis leading to admission for gastric outlet obstruction (GOO). Abdominal radiograph was remarkable for moderate gastric distention. Growth was normal, and body mass index was 15.7 kg/m^2^ (53rd percentile). Vital signs were normal. He was alert with persistent epigastric pain and tenderness, normal bowel sounds, and soft abdomen with no rebound or guarding. Upper endoscopy, with a GIF-60 endoscope using total intravenous anesthesia with propofol, revealed a toy impacted within the pyloric channel (Fig. 1A). The toy was removed with a retrieval net without complications. Further endoscopic evaluation revealed no evidence of ulcers or erosions (Fig. 1B). The patient reported resolution of symptoms and was discharged home the same day.

The incidence of GOO in pediatric patients is ~2–5 per 1000 per year, with the most common cause being idiopathic hypertrophic pyloric stenosis. GOO is an uncommon presentation of foreign body ingestion (FBI). Clinical features of FBI include drooling, abdominal pain, chest pain, emesis, wheezing, stridor, and respiratory distress. When excluding idiopathic hypertrophic pyloric stenosis, the incidence of GOO in children is 1 in 100 000 births.^1,2^ A study examining FBI in young children from 1995 to 2015 found that ~20% of toy ingestions occurred in children ages 3–4 years, predominantly male.^3^ The plastic toy ingested by our patient was a radiolucent blunt object. Removal is nonemergent unless the patient has acute symptoms, respiratory problems, or is unable to manage secretions.^4^ Asymptomatic patients who swallow radiolucent objects have a significantly delayed diagnosis, further increasing the risk of complication.^4^ However, symptoms in our patient prompted urgent removal. Initial evaluation consists of imaging with radiograph which often shows the etiology of symptoms. Other noninvasive strategies such as contrast studies may be needed to evaluate radiolucent objects in the setting of a suspected FBI.^5,6^ FBI should be considered in any young child presenting with acute-onset vomiting of unclear etiology.
